# A two-night polysomnography preliminary study in pregnant women with insomnia: suicidal ideation and nocturnal cognitive arousal prospectively predict objective nocturnal wakefulness

**DOI:** 10.1093/sleepadvances/zpad016

**Published:** 2023-03-07

**Authors:** David A Kalmbach, Philip Cheng, Thomas Roth, Cynthia Fellman-Couture, Chaewon Sagong, Christopher L Drake

**Affiliations:** Division of Sleep Medicine, Thomas Roth Sleep Disorders & Research Center, Henry Ford Health, Detroit, MI, USA; Department of Obstetrics, Gynecology, and Reproductive Biology, College of Human Medicine, Michigan State University, Grand Rapids, MI, USA; Division of Sleep Medicine, Thomas Roth Sleep Disorders & Research Center, Henry Ford Health, Detroit, MI, USA; Division of Sleep Medicine, Thomas Roth Sleep Disorders & Research Center, Henry Ford Health, Detroit, MI, USA; Division of Sleep Medicine, Thomas Roth Sleep Disorders & Research Center, Henry Ford Health, Detroit, MI, USA; Division of Sleep Medicine, Thomas Roth Sleep Disorders & Research Center, Henry Ford Health, Detroit, MI, USA; Division of Sleep Medicine, Thomas Roth Sleep Disorders & Research Center, Henry Ford Health, Detroit, MI, USA

**Keywords:** pregnancy, depression, PSG, sleep, suicide, worry, rumination

## Abstract

**Study objectives:**

Sleep disruption is common in pregnancy, manifesting as insomnia in half of pregnant women as well as increasing objective nocturnal wakefulness across gestation. Despite potential overlap between insomnia and objective sleep disturbances in pregnancy, objective nocturnal wakefulness and its potential contributing factors remain uncharacterized in prenatal insomnia. The present study described objective sleep disturbances in pregnant women with insomnia and identified insomnia-related predictors of objective nocturnal wakefulness.

**Methods:**

Eighteen pregnant women with clinically significant insomnia symptoms (*n* = 12/18 with DSM-5 insomnia disorder) underwent two overnight polysomnography (PSG) studies. Insomnia symptoms (Insomnia Severity Index), depression and suicidal ideation (Edinburgh Postnatal Depression Scale), and nocturnal cognitive arousal (Pre-Sleep Arousal Scale, Cognitive factor) were assessed before bedtime on each PSG night. Unique to Night 2, participants were awakened after 2 minutes of N2 sleep and reported their in-lab nocturnal (i.e. pre-sleep) cognitive arousal.

**Results:**

Difficulty maintaining sleep was the most common objective sleep disturbance affecting 65%–67% of women across both nights, which contributed to short and inefficient sleep. Nocturnal cognitive arousal and suicidal ideation were the most robust predictors of objective nocturnal wakefulness. Preliminary evidence suggested nocturnal cognitive arousal mediates the effects of suicidal ideation and insomnia symptoms on objective nocturnal wakefulness.

**Conclusions:**

Nocturnal cognitive arousal may facilitate upstream effects of suicidal ideation and insomnia symptoms on objective nocturnal wakefulness. Insomnia therapeutics reducing nocturnal cognitive arousal may benefit objective sleep in pregnant women presenting with these symptoms.

**Statement of Significance**

Insomnia symptoms and objective sleep disturbances are common in pregnancy, yet little is known about the relationship between insomnia and objective sleep problems during this period. In our study, pregnant women underwent two nights of polysomnography monitoring, which revealed that difficulty staying asleep was the most common objective sleep problem exhibited during pregnancy. Notably, this observation comports with the most common patient-reported insomnia complaints during pregnancy. Interestingly, insomnia complaints did not reliably predict objective sleep problems. Rather, heightened cognitive activity at night and suicidal thoughts were strong predictors of objective sleep problems. Preliminary evidence suggests that nocturnal cognitive activity may facilitate upstream effects of suicidal thoughts and insomnia symptoms on objective sleep disturbances.

## Introduction

Approximately half of all pregnant women develop insomnia, including clinically significant symptoms or meeting full criteria for the disorder [1–4]. Although scientific investigation of prenatal insomnia has increased substantially over the past 20 years [5], we still know very little about objectively measured sleep disturbances in prenatal insomnia. This gap is critical for two important reasons. First, in the general patient population, insomnia with objective sleep disturbances (e.g. short duration, low sleep efficiency) tends to be more severe and may be more treatment resistant than insomnia without objective sleep disturbances [6–9]. Second, objective nocturnal wakefulness—irrespective of insomnia status—is associated with increased risk for adverse pregnancy- and fetus-related outcomes [10–13]. Thus, identifying contributing factors to objective nocturnal wakefulness in prenatal insomnia would provide insight into wake-promoting processes, which may reveal treatment targets in insomnia therapy to improve patient outcomes and objective sleep for the benefit of mother and child.

### Objective sleep disturbances in pregnancy

A 2020 qualitative review identified fewer than 10 studies that evaluated polysomnography (PSG) sleep macrostructure in pregnant women [14]. Although none of these studies examined known insomnia cases, we can nevertheless consider objective sleep patterns in the overall pregnant population. Garbazza et al. summarized that pregnant women exhibit greater objective nocturnal wakefulness on PSG relative to age-matched nonpregnant women. Specifically, pregnant women exhibited reductions in sleep efficiency and sleep duration due to an increase in wakefulness after sleep onset. Importantly, review of the literature showed that objective nocturnal wakefulness increases across gestation [14], which comports with patient perceptions [3, 15, 16]. However, as none of these reviewed studies focused on pregnant women with insomnia [14], objective sleep in this clinical population remains uncharacterized.

### Contributing factors to objective nocturnal wakefulness

Although no published studies have identified contributing factors of objective nocturnal wakefulness in pregnancy, we can glean contributing factors from the non-perinatal population that may play a similar role in pregnancy. Interestingly, patient-reported nocturnal insomnia symptoms (e.g. prolonged sleep latency and/or wake after sleep onset) are often inconsistent with objective sleep data [6, 17]. Indeed, objective sleep disturbances do not reliably differ between adults with and without insomnia disorder [18]. Moreover, only about half of the insomnia patient population presents with objective sleep disturbances, whereas the other half of insomnia patients exhibit normative objective sleep [8, 9, 19, 20]. Even so, emerging evidence suggests that high cognitive arousal—a central feature of insomnia [21]—appears to serve as a facilitating mechanism between patient-reported insomnia symptoms and downstream consequences including mental health symptoms and objective sleep disturbances [18].

High cognitive arousal refers to heightened cognitive activity—often in the form of perseverative thinking, like worry and rumination—and is associated with insomnia symptom severity and comorbid mental health symptoms, such as depression and suicidal ideation [22–26]. Focus group data from pregnant women emphasize the critical role of cognitive arousal in prenatal insomnia by describing high cognitive arousal as central to their insomnia experience (e.g. having an active mind, intrusive broad and perinatal-specific worries) [22]. Our team recently demonstrated a positive feedback loop in peripartum between insomnia and nocturnal cognitive arousal wherein high cognitive arousal increases insomnia symptoms, which, in turn, increase cognitive arousal and so on [27]. Moreover, we observed that this cycle is depressogenic such that both insomnia and cognitive arousal increase depressive symptoms during pregnancy and postpartum [27]. Yet, the pathogenicity of high cognitive arousal in insomnia may extend beyond depression as rates of suicidal ideation are highest among pregnant women with insomnia and high cognitive arousal (17%) rather than insomnia alone (6%), cognitive arousal alone (7%), or neither (5%) [23, 26]. These data align with frameworks suggesting emotion dysregulation underlies the relationship between insomnia and suicidality [28, 29].

Laboratory data suggest that the harmful effects of cognitive arousal extend beyond clinical symptoms and contribute to objective nocturnal wakefulness as well. In a multinight in-lab study, our team observed that heightened cognitive arousal at night was associated with objective nocturnal wakefulness in a non-perinatal sample of good sleepers and insomnia patients [18]. PSG data showed that individuals with high nocturnal cognitive arousal, relative to those with low arousal at night, exhibited longer sleep latency (58 vs. 21 minutes), longer wake after sleep onset (72 vs. 27 minutes), lower sleep efficiency (71% vs. 87%), and shorter total sleep time (5.7 vs. 7.0 hours). Importantly, the disruptive effect of cognitive arousal on objective sleep is ecologically valid as supported by actigraphy data showing nocturnal cognitive arousal—specifically in this study as ruminating at night—prolongs objective sleep latency at home [30, 31]. Taken together, these data suggest that high cognitive arousal is not only associated with patient perceptions of nocturnal wakefulness but is also associated with objectively measured nocturnal wakefulness via PSG and actigraphy. As pregnant women with insomnia endorse high levels of cognitive arousal [23], it is possible that high nocturnal cognitive arousal may prolong objective nocturnal wakefulness in pregnancy.

### Study objectives and hypotheses

In the present study, we sought to preliminarily describe objective sleep in pregnant women with insomnia and identify predictors of objective nocturnal wakefulness in this patient population. To achieve these objectives, pregnant women with clinically significant insomnia symptoms slept in a sleep center for two nights of PSG monitoring (1 week apart). First, we described objective sleep disturbances in this sample of pregnant women with insomnia. In accordance with objective sleep patterns in the broader pregnant population [14] and with symptoms reported by pregnant women with insomnia [3, 15, 32], we hypothesized that difficulty maintaining sleep (evidenced by prolonged wake after sleep onset on PSG) would be the most common objective sleep disturbance observed and the largest source of overnight total wake time (opposed to sleep latency).

Second, we identified insomnia-related clinical factors that prospectively predict objective nocturnal wakefulness. Specifically, we tested whether nocturnal cognitive arousal, insomnia symptoms, depressive symptoms, or suicidal ideation predicted objective nocturnal wakefulness as measured by PSG. Per a prior prospective PSG and actigraphy studies [18, 30], we hypothesized that higher levels of cognitive arousal at night would predict greater objective nocturnal wakefulness. Also consistent with prior findings [18, 30], we hypothesized that patient-reported symptoms of insomnia and depression would not predict objective nocturnal wakefulness. We evaluated suicidal ideation as an exploratory predictor of objective nocturnal wakefulness due to its previously observed association with nocturnal cognitive arousal during pregnancy [2, 23, 26] and because suicidal ideation is robustly associated with nocturnal wakefulness in the general adult population [33–35].

## Methods

### Study setting and participants

This study was conducted at Henry Ford Health, a large health system in Metro Detroit, MI, USA. We emailed an advertisement for an in-lab sleep study to 1705 pregnant women in our system who were between gestational weeks 14 and 28. We did not include women in the first trimester of pregnancy due to elevated risk for miscarriage, whereas we set 28 weeks as the upper-bound limit to allow for sufficient time to complete the two-night in-lab study before childbirth. A total of 281 women contacted our team. After learning study details, 100 women consented to the study and completed an online survey on demographics, sleep, and other health data for eligibility screening purposes. Data collected during screening are referred to as baseline data in the remainder of the manuscript. Of these 100 women, 99 provided analyzable data. Of these 99 screeners, 23 women endorsed clinically significant insomnia (Insomnia Severity Index [ISI] score ≥11) [2]. Eighteen of these women agreed to participate in the multinight sleep study.

### PSG assessments and schedule

The protocol involved two overnight in-lab PSG studies. The first sleep study was scheduled 1 week after baseline assessment. The second sleep study was scheduled 1 week after the first sleep study. On both nights, patients completed surveys 30 minutes before bedtime, which assessed clinical symptoms over the past week. Afterward, patients were connected to PSG by a registered polysomnographic technologist (RPGST) with all electroencephalography (EEG) impedances <10. Bedtime was scheduled at patient-reported habitual bedtime per baseline assessment data. Wake time was scheduled for 8 hours later.

*Night 1* was the PSG adaptation and screening night. On Night 1, we collected data using EEG, electrooculography, and electromyography with a montage routinely used to rule out sleep disorders other than insomnia disorder, such as obstructive sleep apnea (apnea–hypopnea index [AHI] >5).

*Night 2* was the enhanced assessment night, which included the same EEG monitoring from Night 1, but also an in-lab protocol to assess pre-sleep cognitive arousal. RPGSTs monitored data live starting at lights out. After observing two consecutive minutes of N2 sleep, a research assistant entered the sleep study room to awaken the patient without turning on the lights. The patient then completed a brief online survey on a tablet computer to report levels of cognitive arousal after lights out in the lab. Upon completion, the research assistant left the room so that the patient could reinitiate sleep. This enhanced assessment protocol was developed as an improvement upon our previous in-lab study wherein pre-sleep cognitive arousal levels were rated in the morning upon waking [18], which may have been vulnerable to recall bias.

### Measures

*Objective nocturnal wakefulness*. PSG data were scored by certified RPGSTs affiliated with N2Sleep Diagnostics Inc. and in accordance with the American Academy of Sleep Medicine’s 2012 guidelines [36]. N2Sleep Diagnostics Inc. is unaffiliated with the study team, and PSG scorers were blind to hypotheses and to patient-reported data (e.g. clinical symptoms). PSG metrics included AHI, sleep latency (minutes from lights out to first epoch of sleep), wake after sleep onset (minutes awake post onset), total wake time (sleep latency + wake after sleep onset), sleep efficiency (proportion of time in bed asleep), and total sleep time (sleep duration). For descriptive purposes, sleep latency and wake after sleep onset were considered “prolonged” when >30 minutes, which is consistent with recommended quantitative criteria for insomnia [37]. Specific to Night 2, time to “fall back to sleep” after the enhanced assessment was scored in minutes from when the research assistant left the room until the first epoch of sleep. Because PSG time in bed was fixed to 8 hours, inferential statistics involving sleep efficiency, total wake time, and total sleep time are largely redundant. Even so, we report all three metrics for comprehensiveness and because readers may be interested in different metrics.

*All self-report measures were collected via online surveys hosted by Qualtrics LLC*. At baseline assessment, patients reported sociodemographic information (e.g. age, race, relationship status, annual income), health history (e.g. parity, gestational age, snoring), and clinical symptoms. Patients also completed online surveys in the lab 30 minutes before bedtime on both nights. At baseline, the clinical surveys retained their usual timeframe, whereas the timeframe was adjusted when administered on Nights 1 and 2 to assess symptoms over the past week.

The *ISI* is a 7-item self-report measure of insomnia symptoms over the previous 2 weeks [38]. Scores range from 0 to 28 with higher scores indicating greater symptom severity. A clinical cutoff of ISI ≥11 yields good sensitivity (0.79) and outstanding specificity (0.94) for detecting DSM-5 insomnia disorder in pregnancy [2]. The ISI was administered first at baseline screening, then again 30 minutes before bedtime on Nights 1 and 2.

*DSM-5 insomnia disorder* was survey assessed per patient-reported symptoms in accordance with criteria from the Diagnostic and Statistical Manual of Mental Disorders, 5th Edition (DSM-5). To meet criteria, patients were required to endorse sleep disturbances ≥3 nights/week that result in daytime impairment for at least 1 month. Because we are interested in cases that onset prior to and during pregnancy, we included cases based on a duration of ≥1 month (which aligns more with DSM-IV-TR). This survey assessed DSM-5 insomnia disorder methodology has been previously supported in pregnancy [2].

The *Pittsburgh Sleep Quality Index (PSQI)* is a self-report measure of global sleep disturbances [39]. To minimize redundancy with the ISI at baseline, we only report patient baseline estimates of habitual bedtime (item #1), sleep latency (item #2), sleep duration (item #4), sleep onset insomnia (item #5a, dichotomized such that ≥3 days/week was a positive screen), and sleep maintenance insomnia (item #5b, dichotomized such that ≥3 days/week was a positive screen).

The *Pre-sleep Arousal Scale’s Cognitive factor* (PSASC) measured nocturnal cognitive arousal [40], which is validated in pregnancy [2]. The PSASC consists of eight items capturing heightened cognitive activity at night (e.g. “review or ponder events of the day” and “can’t shut off your thoughts”) and scores range from 8 to 40 with higher scores indicating greater arousal. PSASC scores ≥16 indicate high trait nocturnal cognitive arousal in pregnancy [2]. The PSASC was administered first at baseline screening, then again 30 minutes before bedtime on Nights 1 and 2 to assess arousal over the prior week. To enhance assessment of nocturnal cognitive phenomena, patients were awakened on Night 2 (after 2 minutes of N2 sleep) and reported their nocturnal cognitive arousal symptoms while trying to fall asleep in the lab that night on the PSASC (on this specific assessment, patients were directed to report cognitive experiences in the lab that night while trying to fall asleep). We awakened patients from sleep rather than wait until morning to minimize the effects of sleep on recall of pre-sleep experiences. Moreover, we elected to wake up patients during N2 sleep because individuals awakened during N1 sleep often do not perceive they were asleep.

The *Edinburgh Postnatal Depression Scale (EPDS)* is a 10-item self-report survey of depression and suicidal ideation over the past seven days [41]. Scores range from 0 to 30. A cut-point of EPDS ≥10 detects minor and major depression and is used to identify depression cases in Henry Ford Health women’s health clinics. EPDS item #10 assessed suicidal ideation (*thought of harming myself occurred to me*) such that any endorsement was operationalized as a positive screen; several previous studies have assessed perinatal suicidal ideation using this method [23, 26, 42–44].

### Analysis plan

Descriptive statistics, correlational analyses, and regression models were conducted in SPSS 26 (IBM), whereas mediation analysis was conducted in R 4.2.1. Significance was set at 0.05. We first reported patient characteristics at baseline screening. Next, we reported mean ± *SD* for Night 1 and 2 PSG parameters to characterize objective sleep. We conducted paired samples *t*-tests between Nights 1 and 2 to explore whether objective sleep changed across nights, which may reflect a PSG “first night effect.”

To test hypotheses, we evaluated correlations between PSG sleep parameters and nocturnal cognitive arousal, insomnia, depression, and suicidal ideation separately for Nights 1 and 2. Pearson’s correlations were performed for continuous variables, whereas Point-Biserial correlations were utilized when a continuous variable was correlated with a binary variable (e.g. PSG total wake time with suicidal ideation).

**Figure 1.**
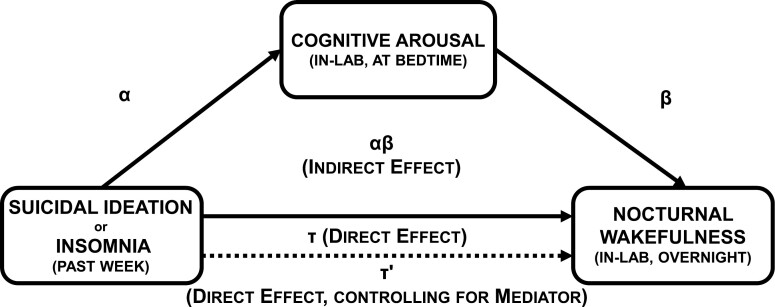
Visual representation of the hypothesized preliminary mediation model wherein cognitive arousal (in the lab) mediates the effects of suicidal ideation or insomnia over the past week on objective nocturnal wakefulness in the lab. *α* represents the effect of suicidal ideation or insomnia on the Mediator (IV ➔ M). *β* represents the effect of the Mediator on the Outcome (M ➔ DV) while controlling for the effects of the IV (suicidal ideation or insomnia). The product of these parameters (*αβ*) represents the indirect effect of suicidal ideation or insomnia on objective nocturnal wakefulness through in-lab nocturnal cognitive arousal (IV ➔ M ➔ DV). If the 95% CI of *αβ* does not overlap with zero, then mediation is inferred.

Based on bivariate results, we conducted two post hoc exploratory mediation models to generate hypotheses for future confirmatory analysis using Night 2 PSG data. We tested two mediation models wherein (Model 1) suicidal ideation or (Model 2) insomnia over the previous week predicted objective nocturnal wakefulness in the lab, which was mediated by nocturnal cognitive arousal in the lab (Figure 1 for visual representation). The direct effect—the tau (*τ*) path—refers to the IV ➔ DV effect. This effect was modeled using linear regression wherein (DV) PSG total wake time was regressed on (IV) suicidal ideation/insomnia over the previous week. Next, we tested the indirect (i.e. mediated) effect. For the alpha path (IV ➔ M), we regressed (M) nocturnal cognitive arousal in the lab on suicidal ideation/insomnia over the previous week. For the beta path (M ➔ DV), we regressed (DV) PSG total wake time on (M) nocturnal cognitive arousal in the lab, while controlling for the previous week’s suicidal ideation/insomnia.

From these regression models, the product of the *α* and *β* parameter estimates represents the indirect (i.e. mediated) effect. The confidence intervals (CIs) of the indirect effect (*αβ*) were estimated using the PRODCLIN method in R 4.2.1 using the RMediation library [45]. This method does not assume a normal distribution, yields asymmetric CIs, and is more accurate than traditional significance tests. If the 95% CI for the indirect effect does not include zero, then significant mediation is inferred. The proportion of the direct effect mediated by the indirect effect can be estimated using this formula: αβ/τ(indirect effect÷direct effect).

## Results

### Patient characteristics at baseline

Seventeen of 18 women completed both overnight sleep studies. Participant ages ranged from 18 to 37 years. All participants were in the 2nd trimester. Most women self-identified racially as non-Hispanic Black (44.4%) or non-Hispanic white (44.4%). See Table 1 for sociodemographic characteristics.

**Table 1.** Patient characteristics at baseline screening

Sample size	18
Age in years (*M* ± *SD*, range)	28.72 ± 4.65, 18–37
Gestational age (*M* ± *SD*, range)	20.44 ± 3.01, 16–26
Race	
Non-Hispanic Black (*n*; %)	8/18; 44.4%
Non-Hispanic white (*n*; %)	8/18; 44.4%
Multiracial	2/18; 11.1%
Poverty (*n*; %)	3/18; 16.7%
BMI (*M* ± *SD*)	28.56 ± 5.55
BMI ≥35 (*n*; %)	2/17; 11.8%
Relationship status (*n*; %)	
Single	2/18; 11.1%
Unmarried, but in a relationship	7/18; 38.9%
Married	9/18; 50.0%
Multiparous (*n*; %)	12/18; 66.7%
Snore (*n*; %)	2/18; 11.1%
DSM-5 insomnia disorder (*n*; %)	12/18; 66.7%
ISI (*M* ± *SD*)	13.78 ± 3.86
Habitual bedtime (median; range)	23:00; 21:00–01:00
Sleep latency, patient-reported minutes (*M* ± *SD*)	32.50 ± 26.19
Sleep onset insomnia ≥3 nights/week	6/18; 33.3%
Sleep maintenance insomnia ≥3 nights/week	10/18; 55.6%
Sleep duration, patient-reported hours (*M* ± *SD*)	6.25 ± 1.15
Short sleep (patient-reported ≤6 h; *n*; %)	11/7; 61.1%
EPDS (*M* ± *SD*)	9.28 ± 4.16
EPDS ≥10 (*n*; %)	10/18; 55.6%
Suicidal ideation (*n*; %)	4/18; 22.2%
PSASC (*M* ± *SD*)	21.50 ± 7.61
PSASC ≥18 (*n*; %)	12/18; 66.7%

BMI, body mass index; *M* ± *SD*, mean and standard deviation; *n*, number of participants; %, percentage of sample. Poverty operationalized as ≤$20 000 in annual income. Sleep latency, sleep onset insomnia, sleep maintenance insomnia, and sleep duration were derived from the Pittsburgh Sleep Quality Index. Suicidal ideation assessed by EPDS item #10 such that any endorsement is a positive screen.

All patients reported clinically significant insomnia symptoms at baseline, and 66.7% (12/18) endorsed criteria for DSM-5 insomnia disorder. Difficulty staying asleep was the chief sleep complaint with 55.6% of patients reporting waking up in the middle of the night or early morning ≥3 nights/week. By comparison, 33.3% of patients reported difficulty falling asleep at least 3 nights/week. At baseline assessment, over half of the sample screened positive for depression (55.6%), two-thirds screened positive for high nocturnal cognitive arousal (66.7%), and four patients (22.2%) endorsed suicidal ideation. All participants denied current antidepressant medication and sleep aid use. See Table 1 for additional sleep- and health-related information.

### Objective nocturnal wakefulness and sleep disturbances

*Night 1: Adaptation*. Only one participant had an AHI ≥5 indicating low frequency of OSA in this sample (Table 2). On Night 1, participants averaged 6 hours and 45 minutes of sleep with a mean sleep efficiency of 84.6%. Prolonged wake after sleep onset (>30 minutes) affected 66.7% of participants, whereas prolonged sleep latency (>30 minutes) affected only 5.6% of women (i.e. one participant). Participants averaged over an hour of total wake time on Night 1. See Table 2 for Night 1 sleep parameters and sleep stage data.

**Table 2.** PSG sleep indices on the adaptation night (Night 1) and baseline night (Night 2)

	Night 1 *n* = 18	Night 2 *n* = 17
AHI (*M* ± *SD*)	1.15 ± 1.43	—
AHI ≥5	1; 5.6%	—
SL (*M* ± *SD*)	13.63 ± 11.24	11.85 ± 8.61
SL ≥30 (*n*; %)	1; 5.6%	1; 5.6%
Fall back to sleep (*M* ± *SD*)	—	10.53 ± 11.14
WASO (*M* ± *SD*)	63.42 ± 80.33	61.65 ± 54.26
WASO ≥30 (*n*; %)	12; 66.7%	11; 64.7%
Sleep efficiency (*M* ± *SD*)	84.55 ± 16.84	85.92 ± 11.90
SE <85%	6; 33.3%	6; 35.3%
Total sleep time (*M* ± *SD*)	405.28 ± 80.86	410.79 ± 60.35
TST <6.5 h	6; 33.3%	5; 29.4%
Total wake time (*M* ± *SD*)	75.65 ± 80.72	73.49 ± 60.14
TWT ≥60 (*n*; %)	8; 44.4%	10; 58.8%
N1 sleep (min; *M* ± *SD*)	18.78 ± 9.49	20.65 ± 11.39
N2 sleep (min; *M* ± *SD*)	246.13 ± 65.44	242.68 ± 47.75
N3 sleep (min; *M* ± *SD*)	56.19 ± 32.58	62.09 ± 42.66
REM sleep (min; *M* ± *SD*)	85.22 ± 39.54	86.16 ± 42.66
REM latency (min; *M* ± *SD*)	106.82 ± 53.52	122.23 ± 88.25

We conducted a series of paired samples *t*-tests for these sleep parameters, which revealed no mean differences between Nights 1 and 2 (all *p* values >.340). Fall back to sleep = minutes to fall back asleep after the nighttime awakening for the enhanced assessment of nocturnal cognitive arousal. SE, sleep efficiency; SL, sleep latency; TST, total sleep time; TWT, total wake time; WASO, wake after sleep onset.

*Night 2: Enhanced assessment*. Once again, prolonged wake after sleep onset was the cardinal feature of objective nocturnal wakefulness affecting 64.7% of participants, whereas prolonged sleep latency affected only 5.6% of participants. Unique to Night 2, participants averaged 10.53 minutes to fall back asleep after being awakened to assess in-lab cognitive arousal. Overall, no sleep parameters significantly differed between Nights 1 and 2, indicating no significant first night effect in this sample (all *p* values >.340). See Table 2 for Night 2 sleep parameters and sleep stage data.

### Do insomnia, depression, cognitive arousal, and suicidal ideation predict objective sleep disturbances?

*Night 1: Adaptation*. A series of zero-order correlations tested bivariate associations between PSG sleep parameters and participant reports of nocturnal cognitive arousal, insomnia symptoms, depression symptoms, and suicidal ideation (Table 3). Analyses revealed that both nocturnal cognitive arousal and suicidal ideation over the previous week prospectively predicted PSG sleep disturbances, including longer wake after sleep onset, lower sleep efficiency, shorter total sleep time, and greater total wake time overnight in the lab.

**Table 3.** Bivariate correlations between PSG indices and patient-reported clinical symptoms for Night 1 (*n* = 18) and Night 2 (*n* = 17)

PSG Night 1	ISI, past week		PSASC, past week		EPDS, past week		SI, past week			
	*r*	*p*	*r*	*p*	*r*	*p*	*r*	*p*		
Sleep latency	0.012	.962	0.198	.432	−0.022	.932	−0.305	.218		
WASO	0.255	.306	0.519	.027	0.210	.403	0.502	.034		
Sleep efficiency	−0.262	.295	−0.565	.014	−0.200	.426	−0.473	.047		
Total sleep time	−0.249	.320	−0.550	.018	−0.200	.406	−0.481	.043		
Total wake time	0.269	.280	0.574	.013	0.209	.406	0.476	.046		

PSG Night 2	ISI, past week		PSASC, past week		EPDS, past week		SI, past week		PSASC, in-lab	
	*r*	p	r	p	r	p	r	p	r	p
Sleep latency	0.449	.071	0.328	.198	0.112	.668	0.221	.395	0.509	.037
Fall back to sleep	0.254	.326	0.342	.180	0.004	.987	0.299	.244	0.541	.025
WASO	0.584	.014	0.560	.019	0.320	.211	0.604	.010	0.677	.003
Sleep efficiency	−0.605	.010	−0.559	.020	−0.320	.211	−0.609	.009	−0.691	.002
Total sleep time	−0.596	.010	−0.594	.012	−3.76	.136	−0.608	.010	−0.708	.001
Total wake time	0.591	.012	0.553	.021	0.304	.235	0.577	.015	0.684	.002

*r* = rho. *p* = significance value. Fall back to sleep = minutes to fall back asleep after the nighttime awakening for the enhanced assessment of nocturnal cognitive arousal. SI, suicidal ideation per EPDS item #10; WASO, wake after sleep onset.

Of note, four participants endorsed suicidal ideation for the week leading up to Night 1 (22.2%). Patients with suicidal ideation experienced an average of 89 more minutes of total wake time overnight compared with the patients without suicidal ideation (*p* = .046). Insomnia and depression did not predict Night 1 PSG sleep.

*Night 2: Enhanced assessment*. We conducted a similar series of correlations between PSG sleep and clinical symptoms (Table 3). Analyses revealed that in-lab nocturnal cognitive arousal was the most consistent predictor of PSG-defined nocturnal wakefulness. Higher in-lab cognitive arousal correlated with longer sleep latency, longer latency back to sleep after the nighttime awakening, longer wake after sleep onset, lower sleep efficiency, shorter total sleep time, and longer total wake time.

Consistent with Night 1, nocturnal cognitive arousal and suicidal ideation over the past week predicted longer wake after sleep onset, lower sleep efficiency, shorter total sleep time, and longer total wake time. Regarding suicidality, we observed that three patients endorsed suicidal ideation in the week leading up to Night 2 (17.6%), which corresponded to 88 more minutes of total wake time overnight relative to those without suicidal ideation that week (*p* = .015). Depression once again did not predict objective nocturnal wakefulness.

Contrary to Night 1 results and our hypotheses, patient-reported insomnia symptoms over the previous week predicted longer wake after sleep onset, lower sleep efficiency, shorter total sleep time, and longer total wake time on PSG.

### Post hoc exploratory: does nocturnal cognitive arousal mediate the effect of suicidal ideation on objective nocturnal wakefulness?

**Figure 2.**
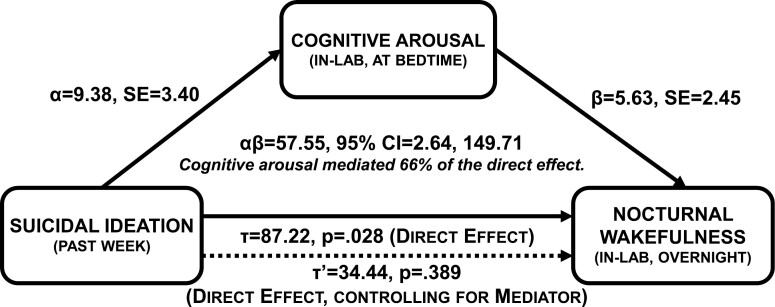
Nocturnal cognitive arousal mediates prospective effects of suicidal ideation on objective nocturnal wakefulness, while controlling for DSM-5 insomnia disorder status. *α* represents the effect of suicidal ideation over the past week on pre-sleep cognitive arousal in the lab (IV ➔ M). *β* represents the effect of pre-sleep cognitive arousal on objective total wake time (M ➔ DV), while controlling for past week suicidal ideation. *αβ* represents the indirect effect of suicidal ideation on objective nocturnal wakefulness as mediated by pre-sleep cognitive arousal.

Nocturnal cognitive arousal and suicidal ideation were the most robust predictors of objective nocturnal wakefulness. Exploration of these data can generate hypotheses for future confirmatory analysis. We propose that patients having suicidal thoughts experience greater cognitive arousal at night, which mediates the prospective effects of suicidal thoughts on objective nocturnal wakefulness. Here, we tested a preliminary model wherein nocturnal cognitive arousal in the lab mediated the effects of suicidal ideation over the past week on objective total wake time overnight in the lab on Night 2 (Figure 2), while controlling for DSM-5 insomnia diagnosis to rule out potential confounding effects between those with vs. without the disorder.

*Tau path (IV* ➔ *DV)*: A linear regression model revealed that patients who endorsed suicidal ideation experienced 87 more minutes awake during Night 2 than patients without suicidal ideation (*b* = 87.22, *SE* = 35.55, *p* = .028) while controlling for DSM-5 insomnia disorder (*b* = 2.42, *p* = .933).

*Alpha path (IV* ➔ *M)*: A linear regression model revealed that past week suicidal ideation predicted in-lab nocturnal cognitive arousal (*b* = 9.38, *SE* = 3.40, *p* = .015) while controlling for DSM-5 insomnia (*b* = 4.79, *p* = .099). *Post hoc descriptive*: The group difference between patients with and without suicidal ideation on in-lab PSASC scores was very large (24.0 ± 8.0 vs. 12.6 ± 4.8; *t*[15] = 3.35, *p* = .004, Cohen’s *d* = 1.73).

*Beta path (M* ➔ *DV)*: A linear regression model revealed that in-lab nocturnal cognitive arousal predicted PSG total wake time such that each 1-point increase on the PSASC corresponded to an additional 5–6 minutes of wakefulness overnight (*b* = 5.63, *SE* = 2.45, *p* = .039) while controlling for suicidal ideation (*b* = 34.44, *p* = .389) and DSM-5 insomnia diagnosis (*b* = −24.56, *p* = .387). *Post hoc descriptive*: Patients with high in-lab nocturnal cognitive arousal (PSASC ≥16) experienced more than an hour longer of total wake time than patients with low in-lab arousal (124.5 ± 88.6 vs. 52.2 ± 27.0 minutes, *t*[15] = 2.65, *p* = .018, Cohen’s *d* = 1.10).

*Indirect path: suicidal ideation* ➔ *nocturnal cognitive arousal* ➔ *objective nocturnal wakefulness*: The PRODCLIN estimate of the indirect effect supported mediation (*αβ* = 57.55, *SE* = 38.67, 95% CI = 2.64, 149.71) whereby suicidal ideation over the past week predicted higher nocturnal cognitive arousal in the lab, which, in turn, prolonged objective total wake time. We estimated that nocturnal cognitive arousal in the lab mediated 66.0% of the effect of suicidal ideation on objective total wake time (57.55/87.22 = 0.660). See Figure 2.

### Post hoc exploratory: does nocturnal cognitive arousal mediate the effect of insomnia on objective nocturnal wakefulness?

**Figure 3.**
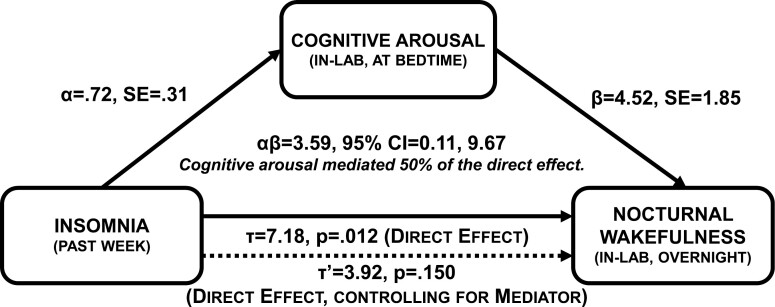
Nocturnal cognitive arousal mediates prospective effects of insomnia symptoms on objective nocturnal wakefulness. *α* represents the effect of insomnia symptoms over the past week on pre-sleep cognitive arousal in the lab (IV ➔ M). *β* represents the effect of pre-sleep cognitive arousal on objective total wake time (M ➔ DV), while controlling for past week insomnia. *αβ* represents the indirect effect of insomnia symptoms on objective nocturnal wakefulness as mediated by pre-sleep cognitive arousal.

Insomnia symptom severity over the previous week predicted objective nocturnal wakefulness on Night 2, which was inconsistent with Night 1 results and evidence from prior research [9, 18]. Even so, it is possible that insomnia symptoms predict objective nocturnal wakefulness, but that this effect is driven by nocturnal cognitive arousal. This interpretation is plausible given that prior research shows insomnia increases nocturnal cognitive arousal [27], coupled with data from the present study showing nocturnal cognitive arousal predicts objective nocturnal wakefulness. Thus, we tested a preliminary model wherein nocturnal cognitive arousal in the lab mediated the effects of past week insomnia symptoms on objective nocturnal wakefulness (Figure 3).

*Tau path (IV* ➔ *DV)*: A linear regression model revealed each 1-point increase on the ISI corresponded to 7 minutes of additional wakefulness overnight (*b* = 7.18, *SE* = 2.53, *p* = .012).

*Alpha path (IV* ➔ *M)*: A linear regression model revealed that insomnia symptoms over the past week predicted in-lab nocturnal cognitive arousal (*b* = 0.72, *SE* = 0.31, *p* = .032).

*Beta path (M* ➔ *DV)*: A linear regression model revealed that in-lab nocturnal cognitive arousal predicted PSG total wake time such that each 1-point increase on the PSASC corresponded to an additional 4–5 minutes of wakefulness overnight (*b* = 4.52, *SE* = 1.85, *p* = .029). In this multivariate model, insomnia symptoms were no longer a significant predictor of total wake time (*b* = 3.92, *p* = .150).

*Indirect path: insomnia* ➔ *nocturnal cognitive arousal* ➔ *objective nocturnal wakefulness*: The PRODCLIN estimate supported mediation (*αβ* = 3.59, *SE* = 2.53, 95% CI = 0.11, 9.67) whereby insomnia over the past week predicted higher nocturnal cognitive arousal in the lab, which, in turn, increased objective total wake time overnight. We estimated that nocturnal cognitive arousal mediated 50.0% of the effect of past week insomnia on objective total wake time during the overnight sleep study (3.59/7.18 = 0.500). See Figure 3.

## Discussion

In a two-night PSG study protocol, we first described objective sleep profiles of 18 pregnant women with clinically significant insomnia symptoms. Important to emphasize is that 12 of these 18 women met DSM-5 criteria for insomnia, thereby reflecting good representation of those with the disorder. Findings from this preliminary study show that sleep maintenance difficulty was the most common objective sleep disturbance observed in pregnant women with insomnia, which reflected patient sleep complaints. Next, we identified nocturnal cognitive arousal and suicidal ideation as robust predictors of objective nocturnal wakefulness. By comparison, patient-reported insomnia symptoms were an inconsistent predictor of objective nocturnal wakefulness, whereas depression was not associated with objective sleep on either night. Evidence from exploratory mediation models preliminarily support indirect effects wherein nocturnal cognitive arousal facilitates prospective effects of suicidal ideation and insomnia symptoms on objective nocturnal wakefulness during pregnancy.

### Patient-reported symptoms and objective sleep profiles in prenatal insomnia

The most common sleep complaint reported by patients was difficulty maintaining sleep. Specifically, 55.6% of pregnant women with insomnia reported difficulty staying asleep ≥3 nights/week, whereas difficulty falling asleep was comparatively less common as reported by 33.3% of women. These results replicate prior studies identifying sleep maintenance as the chief sleep complaint in pregnancy [3, 15, 32].

A novel observation in the present study is the characterization of objective nocturnal wakefulness in prenatal insomnia, which aligned with patient reports. Sleep maintenance difficulty was the most observed objective sleep disturbance in our study such that 65%–67% of pregnant women experienced more than 30 minutes of wakefulness after sleep onset as measured by PSG. By comparison, just 6% of the sample (i.e. one participant) took longer than 30 minutes to fall asleep on either PSG night. Taken together, these data may support an objective basis for sleep maintenance complaints in pregnancy.

Even so, it remains unclear whether objective sleep disturbances differ between pregnant women with and without insomnia. In the non-perinatal population, objective sleep disturbances do not reliably differ between adults with and without insomnia [18, 46], and approximately half of insomnia patients have objective sleep disturbances, whereas the rest have normative objective sleep [8, 9, 19, 20]. Evidence from the present study offers mixed preliminary findings regarding a potential association between patient-reported insomnia and objective nocturnal wakefulness in pregnancy. On Night 1, insomnia over the past week did not predict objective nocturnal wakefulness in the lab, which is consistent with a prior multinight PSG study in the non-perinatal population [18]. However, insomnia symptoms leading up to Night 2 predicted objective nocturnal wakefulness. Reevaluation of this association in a larger sample of pregnant women with and without insomnia would clarify any association (or lack thereof) between insomnia symptoms and objective nocturnal wakefulness during pregnancy.

### Nocturnal cognitive arousal and objective nocturnal wakefulness

Nocturnal cognitive arousal was the most robust predictor of objective nocturnal wakefulness across both PSG nights. These data suggest that faulty cognitive processing—manifested here as heightened cognitive activity in the pre-sleep period—interferes with neurobiological sleep processes rather than merely skewing perceptions of sleep.

Critically, pre-sleep cognitive arousal was not only associated with objective sleep latency, but also to objective difficulty maintaining sleep. These findings are consistent with prior studies in non-perinatal samples evaluating the effects of cognitive arousal on objective nocturnal wakefulness [18, 47, 48]. In a previous two-night PSG study in non-perinatal patients, we observed that adults with high cognitive arousal experienced longer sleep latency (by 36 minutes) and longer wake after sleep onset (by 45 minutes) relative to those with low cognitive arousal, irrespective of insomnia status. Taken together, these data show that cognitive activity in the pre-sleep period disrupts sleep initiation and even continues to disrupt sleep throughout the remainder of the night. This indicates pre-sleep cognitive activity does not dissipate once sleep is initiated, but rather follows the individual into sleep and remains disruptive throughout the night.

### Does cognitive arousal mediate effects of insomnia on objective nocturnal wakefulness?

In the present study, more severe insomnia symptoms predicted greater objective nocturnal wakefulness in the lab on Night 2, manifesting as wakefulness after sleep onset. We must emphasize that this effect was not robust as it was observed on only one of two PSG nights. Moreover, our results also showed that objective nocturnal wakefulness did not differ for patients with and without DSM-5 insomnia disorder. Taken together, these mixed findings align with prior studies showing discrepancy between patient-reported insomnia symptoms and objective nocturnal wakefulness [17, 49].

Nevertheless, this Night 2 effect may have captured insomnia symptoms exerting an indirect influence on objective nocturnal wakefulness via nocturnal cognitive arousal. Indeed, our team and others have shown that insomnia symptoms predict future cognitive arousal symptoms, particularly at night [27, 50, 51]. Moreover, findings from the present study and previous multinight PSG and actigraphy studies show that nocturnal cognitive arousal increases objective nocturnal wakefulness [18, 30, 31]. Taken together, nocturnal cognitive arousal serves as a candidate mechanism for facilitating effects of insomnia on objective nocturnal wakefulness. An exploratory mediation model in the present study supported a pathway wherein insomnia symptoms predicted cognitive arousal during the pre-sleep period, which, in turn, disrupted objective sleep throughout the night.

### Suicidal ideation predicts nocturnal wakefulness in pregnant women with insomnia: nocturnal cognitive arousal may be a mediator

Accumulating evidence from observational studies reveals a close connection between nocturnal wakefulness and suicide-related outcomes, including data showing that suicides often occur at night [33–35, 52]. Although previous studies have conceptualized nocturnal wakefulness as a risk factor for suicide-related outcomes [26, 33–35, 53, 54], our observed prospective effect of suicidal ideation on objective nocturnal wakefulness offers preliminary evidence to suggest that the relationship between nocturnal wakefulness and suicidality may be bidirectional. Prenatal insomnia patients with suicidal ideation experienced 1.5 hours more of total wake time overnight in the lab relative to those without suicidal ideation. Critically, this finding replicated in significance and magnitude across both PSG nights, thereby supporting the robustness of this effect.

In interpreting this finding, we must consider how suicidal thoughts may disrupt sleep. In previous studies, our team has shown that pregnant women with suicidal thoughts report high levels of cognitive arousal at night [23, 27]. In a sample of depressed pregnant women, we observed that suicidal thoughts most commonly occur during periods marked by high cognitive arousal and prolonged wakefulness in the night [26]. Given this context, one potential avenue by which pregnant women with suicidal thoughts experience objective nocturnal wakefulness may be facilitated by the extent to which suicidal thoughts engage nighttime cognitive activity.

This proposed pathway was supported by our exploratory mediation model wherein women with suicidal thoughts spent an additional 1.5 hours awake overnight relative to women without suicidal ideation. We observed that 66% of the effect of suicidal ideation on objective nocturnal wakefulness was mediated by pre-sleep cognitive arousal in the lab. In other words, suicidal ideation over the past week predicted heightened pre-sleep cognitive activity in the lab, which, in turn, disrupted objective sleep throughout the night.

The interplay between wakefulness, cognitive-emotional dysregulation, and suicidal thoughts at night is likely complex and multidirectional. Important to emphasize is that temporal and causal effects are likely quite proximal, occurring between moments within a night. An example of a suicidal ideation ➔ nocturnal cognitive arousal ➔ nocturnal wakefulness pathway: Thoughts of self-harm or suicide increase cognitive activity and cognitive intrusions at night. This increased cognitive activity prolongs nocturnal wakefulness by disrupting sleep initiation and maintenance. Within the same night, prolonged wakefulness offers more opportunity for nocturnal cognitive intrusions and suicidal thoughts, which may, in turn, further prolong wakefulness, and so on. Given the probable multidirectionality of associations among insomnia, cognitive arousal, and suicidal thoughts, suicide-mitigation efforts may benefit from multipronged approaches.

### Clinical implications

Although extant data are somewhat mixed, multiple randomized controlled trials (RCTs) suggest that cognitive-behavioral therapy for insomnia (CBTI) efficacy may be reduced for insomnia patients with objective sleep disturbances (e.g. total sleep time <6 hours) [8, 9, 55, 56]. As an emerging literature implicates nocturnal cognitive arousal as a determinant of objective nocturnal wakefulness [18, 30, 31], it is possible that prenatal insomnia accompanied by high nocturnal cognitive arousal and consequent objective nocturnal wakefulness may signal greater pathology and/or poorer treatment responsivity. Indeed, refractory cognitive arousal—i.e. arousal symptoms that do not change with active treatment—is a robust predictor of CBTI nonresponse in pregnancy [57]. Future studies should evaluate whether objective sleep disturbances play a role in treatment resistance linked to refractory cognitive arousal.

Important to highlight is that CBTI yields minimal effects on cognitive arousal despite its cognitive component [57–60]. Further, behavioral sleep strategies—even when successful—appear limited in ability to quell nocturnal cognitive arousal or its harmful effects. Although sleep may seemingly offer a reprieve from a highly active mind, data suggest that sleep alone may not quiet an active mind. Rather, an active mind disrupts physiologic sleep throughout the night (per present study data and prior studies [18, 30]) and can even influence the content of REM dreaming [61].

As nocturnal cognitive arousal contributes to objective nocturnal wakefulness, enhancing insomnia therapy with components that better reduce nighttime cognitive activity may optimize patient outcomes [57, 62, 63] and may even benefit objective sleep in insomnia [9]. Growing evidence suggests that imbuing insomnia therapy with mindfulness components may enhance alleviation of cognitive arousal [64, 65]. Importantly, focus groups of pregnant women with sleep problems have expressed strong interest in mindfulness-based interventions to improve sleep and reduce both general and perinatal-specific perseverative thinking, suggesting possibility of high patient uptake [22]. Although clinical trial data on mindfulness-based interventions for prenatal insomnia have not yet been published, available data show that everyday mindfulness is inversely related to nocturnal cognitive arousal in pregnancy [66], and that mindfulness training protects pregnant women against the harmful effects of poor sleep on stress [67].

*Insomnia therapy for suicide-risk mitigation*. In an RCT, we previously observed that digital CBTI may reduce suicidal thoughts in pregnant women with insomnia [59], which is consistent with a growing number of RCTs supporting CBTI effects on suicide-related outcomes in the non-perinatal population [54, 68–70]. Mechanistic exploration in the non-perinatal patient population shows that suicidolytic effects of CBTI are driven by alleviation and remission of insomnia symptoms [54, 70]. However, reducing cognitive arousal has been proposed as an important treatment target to reduce suicidal thinking [71]. Unfortunately, CBTI undertreats cognitive arousal symptoms in pregnant women with insomnia [57, 72]. Insomnia therapies that effectively reduce insomnia symptoms while also alleviating cognitive arousal may optimize suicide-related outcomes in pregnant women with insomnia.

### Methodological limitations

In the present study, the most notable methodological limitation is the lack of a good-sleeping control group, which precludes determination of whether objective sleep differs between pregnant women with and without insomnia. In the non-perinatal population, objective sleep does not reliably differ between adults with and without insomnia [18]. However, it is possible that the physiologic changes in pregnancy that contribute to sleep disruptions may influence patient reports of insomnia, thereby lending insomnia complaints—at least for some patients—an objective foundation. To illustrate: Pregnant women who objectively wake up more frequently due to discomfort have *more opportunity* for having difficulty falling back asleep, which may contribute to patient-reported insomnia complaints.

Another limitation is the small sample size of 18, which limited ability to test more complex models. In addition, we likely captured restricted range in symptoms of insomnia and cognitive arousal due to the nature of sampling women with prenatal insomnia. As such, we frame our mediation models as preliminary and in need of replication. Even so, we emphasize strongly that our results are consistent with the extant literature on objective sleep disturbances and their associations with cognitive arousal, insomnia, and suicide-related factors. Moreover, outcomes were largely replicated across multiple PSG nights, thereby supporting the reliability of our study findings. Future studies would benefit by replicating these findings in a larger sample that includes pregnant women with and without insomnia.

Regarding our enhanced assessment of in-lab cognitive arousal, we are not aware of a protocol for waking participants from sleep in a PSG study to capture pre-sleep cognitive activity. We elected to wake up patients during N2 sleep rather than N1 sleep since individuals awakened during N1 sleep often do not perceive they were asleep. However, it is unclear whether this method is preferred over other potential wake-points such as the first epoch of scored sleep.

A final limitation is that certain pregnancy-related factors that may disrupt sleep were not assessed, including nausea, heartburn, urinary frequency, and backpain [73]. It is possible that these symptoms contribute to objective nocturnal wakefulness in prenatal insomnia, and that they may even interact with cognition (e.g. experiencing heartburn at night leads to ruminating on heartburn and/or sleep difficulties, which disrupts physiologic sleep processes). Future studies on objective sleep during pregnancy should capture these potential contributing factors.

## Conclusions

Objective sleep data show pregnant women with insomnia exhibit inefficient and short sleep due to increased wake after sleep onset. Heightened cognitive activity at night (e.g. worry, rumination) disrupts both sleep latency and sleep continuity during pregnancy. Suicidal thoughts and insomnia symptoms may yield upstream influences on objective nocturnal wakefulness, but these effects appear to be mediated by cognitive arousal at night. Future studies are needed in four important areas: (1) Replicate these findings in a larger sample including pregnant with and without insomnia, which will clarify prospective associations among nocturnal cognitive arousal, suicidal ideation, insomnia symptoms, and objective nocturnal wakefulness. (2) Compare objective sleep between pregnant women with and without insomnia. (3) Evaluate the potential role of objective sleep disturbance in treatment responsivity during pregnancy. And finally, (4) enhance insomnia therapies to better address nocturnal cognitive arousal and objective nocturnal wakefulness.

## Data Availability

The data underlying this article will be shared on reasonable request to the corresponding author.
